# Transcranial Direct Current Stimulation to Provide Neuroprotection and Enhance Cerebral Blood Flow in Stroke: A Comprehensive Review

**DOI:** 10.3390/medicina60122061

**Published:** 2024-12-14

**Authors:** Muhammed Enes Gunduz, Melike Kocahasan, Zafer Keser

**Affiliations:** 1 Department of Neurology, University of Massachusetts Chan Medical School, Worcester, MA 01655, USA; muhammed.gunduz@umassmed.edu; 2 School of Medicine, Koc University, Istanbul 34450, Turkey; mkocahasan20@ku.edu.tr; 3 Department of Neurology, Mayo Clinic, Rochester, MN 55905, USA

**Keywords:** stroke, neuromodulation, tDCS, cerebral blood flow, neuroprotection

## Abstract

Stroke remains a leading cause of global disability and mortality despite advancements in acute interventions. Transcranial direct current stimulation (tDCS), a non-invasive neuromodulation technique, has primarily been studied for its effects on cortical excitability, with limited exploration of its neuroprotective and hemodynamic benefits. This review examines the role of tDCS in stroke, with a focus on neuroprotection in acute settings and cerebral blood flow (CBF) modulation in both acute and chronic phases. tDCS offers rapid, localized delivery to salvageable ischemic tissue, exerting pleiotropic effects that address a broader spectrum of pathological processes compared to pharmacological agents. Cathodal tDCS shows promise in acute ischemic stroke for neuroprotection in small-scale clinical studies, enhancing CBF and promoting vessel recanalization, while anodal tDCS demonstrates stronger effects on CBF, particularly in chronic stroke and hypoperfusion cases. Bihemispheric stimulation may offer additional benefits, with evidence suggesting a dose-dependent relationship between stimulation parameters and therapeutic outcomes. Further research is warranted to optimize stimulation protocols, evaluate safety and feasibility, and explore the potential of tDCS to promote neuroplasticity and functional recovery across different stroke populations and stages. By addressing these gaps, tDCS could emerge as a valuable adjunctive therapy in stroke management, complementing current interventions and expanding therapeutic windows.

## 1. Introduction

Stroke remains a leading cause of disability worldwide despite recent developments in hyperacute interventions and the growing field of neurorehabilitation therapies [1]. Although recent clinical trials have expanded the use of intravenous thrombolytic therapies and endovascular interventions (i.e., expanding the therapeutic window and patient eligibility) for acute ischemic stroke, or introduced new treatments like minimally invasive hematoma evacuation to improve outcomes in hemorrhagic stroke, the majority of patients that experience stroke are still not eligible for acute interventions due to various reasons [2,3]. Furthermore, even with many promising advances in the field of neurorehabilitation [4], achieving functional independence is limited to only a quarter of patients undergoing these interventions.

To further improve outcomes, many pharmacological therapies have focused on neuro- and vasoprotection in acute stroke, aiming to preserve neuronal cells and the neurovascular unit [5,6]. Neuroprotective therapies aim to protect the ischemic penumbra, especially for patients requiring transfer to other hospitals, those not eligible for hyperacute interventions, or those who experience failed reperfusion. In addition, such therapies aim to prevent reperfusion injury. However, many human studies have failed to demonstrate significant benefits, facing challenges in delivering therapeutic agents directly and effectively to ischemic tissue, which is hindered by altered hemodynamics, reduced perfusion, and blood–brain barrier impairment.

Transcranial direct current stimulation (tDCS) involves delivering a steady, low-amplitude direct current to the brain through electrodes placed on the scalp. The current effectively penetrates the scalp and reaches underlying brain tissue, providing a sub-threshold stimulus that modulates neuronal membrane potentials, thus affecting cortical excitability and the likelihood of neuronal firing [7]. Typically, two forms of tDCS are employed: anodal stimulation, which enhances cortical excitability; and cathodal stimulation, which reduces it. When applied with sufficient duration and intensity, tDCS can induce long-term potentiation or depression, leading to lasting clinical effects. tDCS influences a broad area of the cortex, which helps to engage additional brain regions relevant to the target area as well as non-neuronal tissues like glial cells and blood vessels (Monai 2016, *Nature Communications*) [8]. Furthermore, tDCS is generally safer, easier to administer, and more accessible. Key study parameters include the electrode placement (montage or target area), electrode size (affecting current density), current strength, and the duration and frequency of application. When appropriately screened, tDCS does not produce irreversible adverse effects; common minor side effects are temporary headaches, skin redness, and sensations of tingling or itching during current ramping [9,10]. Across over 1000 subjects receiving over 33,000 tDCS sessions, there were no reported serious adverse events [9]. The US FDA considers tDCS a non-significant-risk intervention. tDCS is a subthreshold stimulation that is far below the seizure induction threshold and considered non-epileptogenic [11]. Similarly, no other serious adverse events have been shown in tDCS studies in stroke populations and there is no evidence of increased risk in patients with large vessel occlusion [12]. tDCS provides further advantages with inexpensive equipment, easy administration, portability, an ability to be used in combination with other therapies, and the possibility of home-based treatment [13,14,15]. tDCS has primarily been utilized to enhance recovery in patients with subacute-to-chronic stroke as an adjuvant therapy, where its effects on cortical excitability and plasticity are leveraged to support motor, language, and cognitive rehabilitation [4].

tDCS can be delivered quickly, directly, and locally to salvageable ischemic tissue, unaffected by the reduced perfusion associated with occluded vessels. Moreover, while pharmacological agents commonly target limited components of ischemic pathways, tDCS exerts pleiotropic effects that target a broader spectrum of pathological processes. This review focuses on tDCS and its potential in acute and chronic stroke, with specific attention to its neuroprotective and hemodynamic mechanisms.

## 2. Neuroprotective Effects of tDCS in Acute Stroke

The pathophysiology of ischemic stroke involves a cascade of detrimental events, including excitotoxicity, oxidative stress, and inflammation, which lead to irreversible neuronal injury. A primary focus of neuroprotective strategies is attenuating excitotoxicity by reducing glutamate release and preventing calcium overload in neurons. The effects of tDCS on these mechanisms in suppressing excitotoxicity and inflammation have been studied with multiple different techniques and targets, including anodal, cathodal, or pulsed stimulation to the ischemic zone, as well as deep brain stimulation targeting remote deeper structures such as fastigial nucleus stimulation, subthalamic vasodilator area stimulation, and dorsal peri-aqueductal gray stimulation [16]. A meta-analysis including 21 preclinical studies assessed the overall effects of electrical stimulation for neuroprotection and compared the impact of these different stimulation strategies. This meta-analysis showed a significantly reduced infarct volume by 37% in rats receiving electrical stimulation with significant treatment effects of different stimulation strategies. For infarct zone hemispheric stimulation, the cathodal stimulation showed a greater impact (27%) in infarct volume reduction, while there was less or no impact of anodal or pulsed stimulation. They also showed a significant impact of deep brain stimulation in all of the remote zones mentioned above, ranging from 45 to 52% reduction. These findings were replicated similarly in another meta-analysis [17]. This evidence pointed out the possible clinical translation of mainly cathodal tDCS stimulation over the ipsilesional infarct zone as a potential neuroprotective approach, given that the major limitations of stimulation of other remote deeper structures with invasive procedures in acute stroke settings make it unfeasible.

The effect of cathodal tDCS was attributed to suppressing excitotoxicity through modulation of the N-methyl-D-aspartic acid (NMDA) receptors, resulting in the downregulation of glutamate-induced excitotoxic pathways, which inhibits peri-infarct depolarizations, a critical driver of infarct expansion. Furthermore, cathodal tDCS has demonstrated the ability to reduce inflammatory processes within the ischemic penumbra. Animal models have shown that electrical stimulation dampens the release of pro-inflammatory cytokines and reduces apoptosis through the inhibition of caspase-3 activation and blockade of microglial and astrocytic activation. By modulating both neuronal and inflammatory pathways, tDCS provides a broader neuroprotective effect compared to pharmacological agents that often target single molecular pathways [18].

Another key target for neuroprotective interventions is to preserve the ischemic penumbra, the brain tissue at risk of infarction but still viable for salvage if reperfusion occurs within a critical time window. Electrical stimulation has been shown to enhance penumbral protection by the mechanisms explained above, such as stabilizing neuronal membranes, reducing excitotoxicity, and maintaining cellular homeostasis. By “freezing” the penumbra through its neuroprotective effects, electrical stimulation may extend the therapeutic window for reperfusion therapies, allowing more time for interventions like thrombectomy. This may be particularly relevant in patients who present outside the conventional therapeutic window or those requiring transfer to specialized centers for treatment.

Clinical evidence of neuroprotection is still limited but growing. Proof-of-concept studies on the feasibility, safety, and tolerability of cathodal transcranial direct current stimulation in acute ischemic stroke have provided preliminary insights into its potential as a non-pharmacologic treatment for neuroprotection and cerebral blood flow enhancement. In the STICA trial, researchers assessed 45 patients with acute ischemic stroke and found that the intervention was both feasible and well-tolerated, with no major adverse effects [19]. While there was no statistically significant difference in infarct growth between the cathodal tDCS and sham groups, a trend toward reduced infarct size was observed in patients with moderate-to-severe stroke and large vessel occlusions. Similarly, the TESSERACT trial demonstrated promising results for acute ischemic stroke intervention, especially in achieving higher rates of penumbral tissue salvage and alleviating hypoperfused regions in patients receiving active treatment versus those in the sham group. This effect was accompanied by a significant enhancement of relative cerebral blood volume (CBV) and an increased rate of early vessel recanalization post-stimulation in active patients [20]. These early findings underscored the value of cathodal tDCS in augmenting standard stroke treatment by potentially limiting infarct growth through neuroprotective mechanisms and enhancing perfusion to the ischemic regions. These trials establish a foundation for further exploration of cathodal tDCS in larger, multicenter trials to evaluate its efficacy in acute stroke intervention.

Two primary mechanisms likely contributed to these observed effects: (i) the direct protective impact on ischemic tissue through its anti-excitatory, anti-apoptotic, and anti-inflammatory actions, thus promoting cytoprotection; (ii) the electrical field from tDCS may enhance collateral pathways, thereby facilitating retrograde reperfusion and improving orthograde blood flow to ischemic regions [20]. Recently, we identified two completed registered studies on ClinicalTrials.gov investigating tDCS in acute stroke patients. A randomized, double-blind, placebo-controlled trial conducted in the Czech Republic aimed to evaluate the effects of cathodal tDCS in patients with disabling acute stroke caused by occlusion of the internal carotid artery or middle cerebral artery and the presence of salvageable penumbra. These patients were not eligible for endovascular or intravenous thrombolytic therapies (NCT04801446). The study employed six dosing tiers, with increasing stimulation intensity and duration, similar to the TESSERACT study. Its primary focus was on safety, tolerability, and feasibility, with symptomatic intracranial hemorrhage serving as the primary safety outcome. Secondary and exploratory outcomes included clinical and imaging efficacy metrics, such as the modified Rankin Scale (mRS), infarct growth, and quality-of-life measures.

Another study, conducted in the Republic of Korea, investigated the effects of either anodal stimulation over the affected hemisphere or cathodal stimulation over the unaffected hemisphere compared to sham tDCS (NCT04938076). The study enrolled patients with acute stroke involving the corticospinal tract who presented with motor weakness within 48 h of symptom onset. Patients who received intravenous thrombolysis or endovascular therapy were excluded. The primary outcome was the Fugl–Meyer Motor Scale, which quantifies motor deficits after stroke, while the secondary outcome included the mRS. The results of both studies are not yet available.

## 3. Effects of tDCS on Cerebral Blood Flow

**Figure 1:**
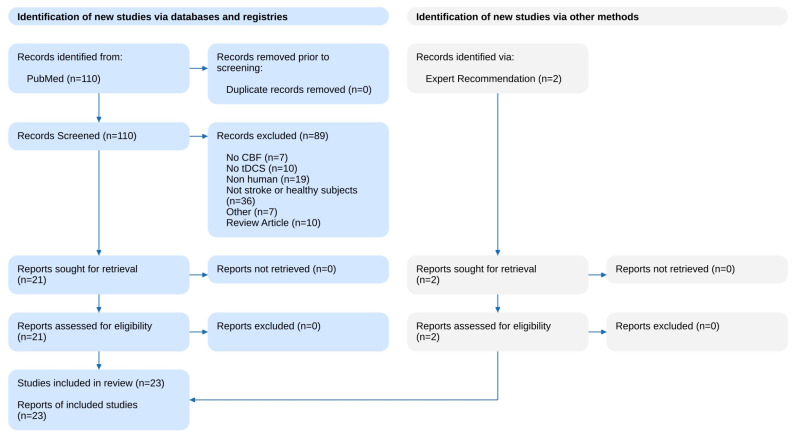
The literature search flowchart.

We conducted a systematic search of the PubMed database using Nested Knowledge systematic review software with the following search string: (tdcs OR transcranial direct current stimulation) AND (cerebral blood flow OR CBF). The search included studies published between January 2000 and December 2024. To supplement the database search, we performed a manual search and reviewed the reference lists of included papers to identify additional relevant studies Inclusion criteria included original articles assessing direct or indirect measures of CBF in stroke patients and/or healthy volunteers. Exclusion criteria included animal studies, duplicate records, and review articles. Two authors (MEG and MK) independently screened the studies, with any discrepancies resolved through discussion and consensus among all authors after reviewing the full text of the articles. Figure 1 summarizes the results of the search and screening process.

The cerebral blood flow response to various tDCS protocols has been extensively studied in animal models and healthy participants, employing a range of stimulation parameters such as target region, intensity, montage, polarity, and dosage. Different methodologies—including arterial spin labeling (ASL) via magnetic resonance imaging (MRI), H215O positron emission tomography (PET), functional near-infrared spectroscopy (fNIRS), cerebral vasomotor reserve, and vessel-specific blood flow velocity assessed by transcranial color-coded sonography—have been used to evaluate CBF changes. These findings are detailed in Table 1.

In a study by Lang et al. (2005) involving 16 healthy participants, both anodal and cathodal tDCS were shown to induce comparable increases in CBF—irrespective of the polarity of regions beneath the electrodes (frontopolar and left motor cortex (M1))—when compared to sham stimulation [21]. These effects extended beyond the areas of stimulation, reaching widespread brain regions including subcortical structures, the caudal part of the anterior cingulate cortex, right parieto-occipital junction, superior temporal sulcus, cerebellum, and the contralateral M1. Notably, the CBF changes in remote areas exhibited polarity-dependent patterns, with anodal tDCS affecting dorsal cerebellar regions and cathodal stimulation impacting ventral areas. Another study on healthy participants, using an interleaved tDCS-on/tDCS-off protocol while simultaneously acquiring ASL images, also demonstrated the polarity-dependent effects: both anodal and cathodal stimulations led to significant CBF increases during stimulation, though anodal effects were more pronounced (17.1% vs. 5.6%) [26]. Additionally, while anodal stimulation resulted in sustained CBF elevation post-stimulation, cathodal stimulation showed a decline. With the exception of a study using only a brief 30-second tDCS duration (compared to other studies with 10–30 min) [31], all studies reported similar CBF responses and prolonged post-stimulation effects lasting from 30 to 60 min to up to 24 h. These findings indicate that tDCS has the potential to produce sustained and network-wide alterations in cerebral hemodynamics, extending beyond the regions immediately beneath the electrodes. 

Several studies have explored the dose-dependent effects of tDCS on cerebral blood flow. Jamil et al. (2020) conducted a study with 29 healthy volunteers using varying current intensities (0.5, 1, 1.5, and 2 mA) and found that 2 mA elicited the most pronounced alterations in CBF compared to lower intensities. Moreover, the effects of 2 mA were sustained for longer, across the entire two-hour post-stimulation evaluation period. Another study corroborated these findings, demonstrating a significant correlation between higher current intensity (ranging from 1 to 1.7 mA) and greater CBF changes, although the polarity effect appeared to exert a stronger influence on CBF modulation than the dose itself [26]. Building on this evidence, additional research examined even higher intensities, comparing 2 mA to 4 mA, based on emerging data suggesting that higher intensities (e.g., 3 or 4 mA) may enhance efficacy [29,42]; this study reported greater regional CBF increases with the 4 mA intensity, further supporting the potential benefits of higher-dose tDCS.

The duration of stimulation also plays a critical role in determining the overall dose effect. While the impact of stimulation duration on CBF was not systematically tested in the studies we identified, one study observed that longer stimulation times (e.g., 15 min compared to 10 min) resulted in more prolonged and sustained CBF changes [23]. These findings highlight the importance of optimizing both the intensity and duration of tDCS to maximize its hemodynamic effects.

Most of the studies focused on different polarity and dose effects, however, different tDCS montages were tested to a limited extent. A study by Shinde A. et al. (2021) provided crucial insights into how different tDCS montages, particularly bihemispheric stimulation, influence CBF with a distinct pattern. The study compared unihemispheric and bihemispheric stimulation with sham, 2 ma, and 4 mA intensities with a concurrent tDCS-MRI protocol [29]. Using a 4 mA dose, the bihemispheric montage induced widespread and bilateral increases in CBF, including significant changes in peri-rolandic and frontomesial regions. Unlike the unihemispheric montage—primarily affecting the peri-rolandic region under the anodal electrode—bihemispheric stimulation leveraged interactions between both hemispheres, resulting in additional activation in the cathodal hemisphere and functionally connected regions, which pointed out that in higher intensity bihemispheric stimulation, the cathodal electrode may induce excitatory changes rather than the traditionally presumed inhibitory effects. Finally, Kolamjai W. et al. (2022) compared three different montages; although they did not show changes in cerebral blood flow velocity, measured with transcranial Doppler, the bihemispheric stimulation led to significantly greater clinical improvement compared to other montages [41]. These effects suggest that higher doses of bihemispheric tDCS enhance interhemispheric communication and reduce transcallosal inhibition, which could amplify the neuromodulatory impact beyond localized areas, underscoring the importance of montage selection, particularly in therapeutic applications where network-wide hemodynamic effects might be beneficial.

The direct effects of tDCS on specific vessel velocity have also been investigated as a measure of CBF. Transcranial color-coded duplex sonography, also referred to as transcranial Doppler (TCD), provides a means to assess cerebral vasomotor reserve, offering insights into the ability of cerebral arteries to adjust their diameter in response to stimuli. Giorli et al. (2014) reported polarity-specific changes in cerebral vasomotor reserve with 1 mA tDCS over the motor cortex, demonstrating a decrease in mean arterial flow in the middle cerebral artery (MCA) during anodal stimulation, while cathodal stimulation with increased flow, compared to the sham, did not produce significant effects [36]. However, these findings were not replicated in subsequent studies. Stefano et al. (2022) observed no statistically significant changes in MCA velocity measured via TCD in a small sample of healthy volunteers in response to 1, 2, or 3 mA anodal or cathodal tDCS over the right temporo-parietal junction [35]. Similarly, a study in 20 chronic stroke patients found no changes in MCA blood velocity during 2 mA anodal tDCS over the M1, although cathodal stimulation was not tested [40]. An alternative approach was used in another study for assessing vessel-specific CBF changes using phase-contrast MRI with time-of-flight angiography as a reference, aiming to measure blood velocity in the four major cerebral vessels, including the bilateral internal carotid and vertebral arteries [34]. Using this approach, global increases in cerebral blood flow were observed during tDCS and persisted after the stimulation period. Notably, the study used a bihemispheric montage, with the anodal electrode positioned over the left dorsolateral prefrontal cortex and the cathodal electrode over the right. These findings highlight the potential of tDCS to induce global and sustained hemodynamic effects, which may vary depending on stimulation montage and polarity.

**Figure 2:**
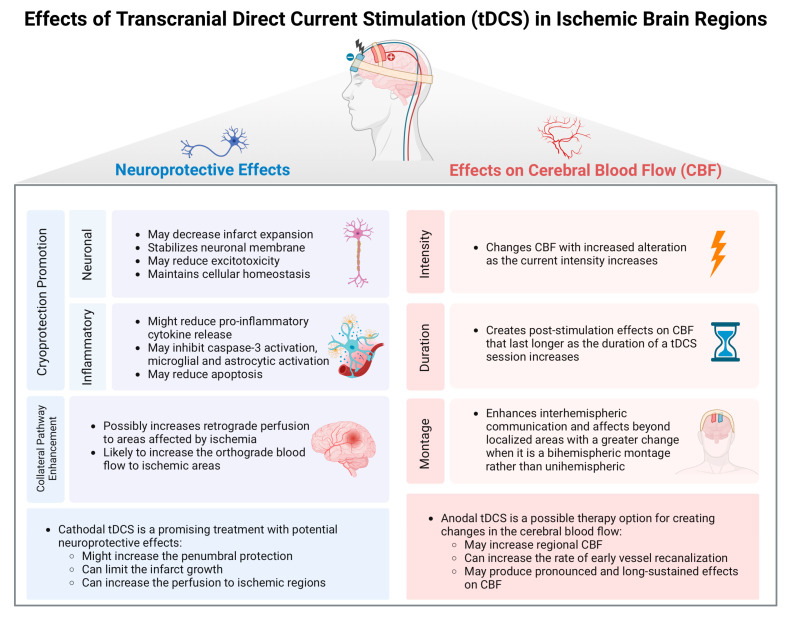
Effects of tDCS in ischemic brain regions.

While most studies assessing the effects of tDCS on CBF have been conducted on healthy volunteers, only a limited number have focused on stroke patients. In chronic stroke patients, Dutta et al. (2015) investigated the effects of anodal tDCS using near-infrared spectroscopy (NIRS) [39]. Their findings demonstrated rapid alterations in regional oxy- and deoxyhemoglobin levels, suggesting blood flow changes in response to electrical stimulation. Cathodal tDCS effects on CBF were not specifically tested in chronic stroke patients in the literature. Additionally, Iyer et al. (2019) specifically assessed CBF using TCD in chronic stroke patients, although the primary aim of the study was to explore the potential influence of CBF changes on tDCS response [40]. While they were unable to detect any changes in cerebral blood flow velocity within the MCA of the lesioned hemisphere, they observed that tDCS-responders exhibited distinct baseline characteristics with lower corticomotor excitability (measured via transcranial magnetic stimulation), reduced cerebral blood velocity, and higher vascular resistance. These findings suggest that, in chronic stroke patients, baseline electrophysiological and vascular characteristics may significantly influence the response to tDCS. Further research is warranted to evaluate these biomarkers and their predictive value, with the ultimate goal of identifying responders and individualizing tDCS treatment options to individual patient profiles. The effects of tDCS in ischemic brain regions were summarized in Figure 2.

## 4. Neuroimaging Biomarkers of CBF

Traditionally, PET was considered the gold standard for measuring CBF. PET utilizes radiolabeled tracers, such as [^15O]-water, to measure regional blood flow by detecting gamma rays emitted during the decay of the tracer [43]. This allows for the quantification of CBF with high accuracy. However, PET is associated with several limitations, including a complex procedural setup, the need for invasive arterial sampling, high costs, limited availability, and its primary use in research settings rather than clinical practice [44].

Earlier studies investigating cerebral hemodynamics also utilized fNIRS, which indirectly measures cerebral blood flow by quantifying changes in oxygenated and deoxygenated hemoglobin. While useful, fNIRS lacks the spatial resolution and quantification capacity of advanced neuroimaging techniques.

In recent tDCS studies, pseudo-continuous arterial spin labeling (pcASL) and blood-oxygenation-level-dependent (BOLD) functional MRI (fMRI) have become preferred methods for assessing CBF. ASL, in particular, offers several advantages over PET. It is a non-invasive MRI technique that uses magnetically labeled arterial blood water as an endogenous tracer to quantify cerebral perfusion. ASL does not require contrast agents, is widely available, and is increasingly used in clinical settings. A recent study concluded that ASL is a promising, robust, accurate, and reproducible method for measuring CBF, further solidifying its role in contemporary research and clinical applications [44]. Although the use of concurrent tDCS with MRI appears to be the most robust method for evaluating neurophysiological effects during and immediately after stimulation, it poses significant challenges. The logistical complexity, high costs, and potential incompatibility of MRI with acute clinical settings limit its feasibility, especially in emergency or routine practice.

TCD is another method frequently employed to measure cerebral hemodynamics. TCD indirectly measures blood flow by assessing velocity within major cerebral arteries [45]. It is non-invasive, portable, and provides real-time data, making it a practical tool in various clinical scenarios. However, TCD is limited to large vessels, does not offer direct quantification of CBF, and many studies have failed to detect CBF changes using this method, raising concerns about its sensitivity for the subtle flow alterations induced by tDCS.

Finally, computed tomography (CT) and MRI perfusion imaging are commonly used in clinical practice, particularly to identify salvageable penumbra in patients with acute stroke. While these techniques are valuable for patient selection in hyperacute neuroprotection studies, they are rarely employed as primary outcome measures to evaluate the effects of tDCS interventions on cerebral perfusion [37,38]. This reflects a focus on their diagnostic utility rather than longitudinal assessment in interventional research.

In summary, while traditional methods like PET and fNIRS laid the foundation for CBF measurement, recent advancements have favored non-invasive, accessible techniques such as pcASL. However, the most reliable outcomes are likely achieved with MRI-based approaches, albeit with limitations in clinical applicability. TCD and perfusion imaging remain valuable tools in specific contexts, although their utility as primary outcome measures for tDCS studies is restricted.

The findings from these studies included in our review are constrained by several limitations. The current literature on the neuroprotective and hemodynamic effects of tDCS in stroke populations is limited, requiring further investigation to establish its efficacy across diverse patient populations and characteristics. Many studies to date have been conducted with relatively small sample sizes, which may limit the generalizability of their findings. Additionally, there is considerable variability in stimulation protocols, including polarity, intensity, duration, and montage as well as differences in outcome measurement techniques. These methodological inconsistencies highlight the need for standardized protocols and larger, well-controlled clinical trials to validate the therapeutic potential of tDCS in stroke care.

## 5. Conclusions/Future Directions

Cathodal tDCS shows promise as a neuroprotective intervention in ischemic stroke patients with salvageable penumbra. In addition to its neuroprotective effects, preliminary evidence suggests that cathodal tDCS may enhance CBF and promote early vessel recanalization following stimulation. While the available studies are limited, bihemispheric stimulation appears to offer greater benefits, and evidence supports a dose-dependent effect of tDCS on outcomes. To address variability in tDCS responses, further advancements are needed in individualized approaches, including novel electrode montages and electric field modeling. These methods aim to deliver more precise and focal stimulation, resulting in more consistent therapeutic effects [46,47]. By reducing dose variability at the cortical level and enabling personalized dosing, these innovations have the potential to enhance the efficacy and reliability of tDCS in clinical applications.

In healthy volunteers, anodal tDCS has demonstrated a stronger effect on enhancing blood flow compared to cathodal tDCS. This effect has also been observed in small studies involving chronic stroke populations. However, further research is needed to determine how these findings can be effectively translated into acute and chronic stroke settings.

Future research should investigate the potential of tDCS to promote recovery in chronic stroke patients by enhancing CBF and supporting neuroplasticity. Particular focus should be given to exploring anodal tDCS in patients with chronic hypoperfusion caused by atherosclerotic or non-atherosclerotic vasculopathies, assessing its potential to improve neovascularization and recovery. If proven effective, tDCS may be an adjunctive therapy for revascularization interventions such as bypass surgeries.

Additionally, studies are needed to evaluate the neuroprotective effects of tDCS in acute hemorrhagic stroke, particularly in terms of its safety, feasibility, and ability to mitigate secondary brain injury. Future research on these topics could provide valuable insights into the broader therapeutic application of tDCS across various stroke populations and stages.

## Figures and Tables

**Table 1:** tDCS studies on cerebral blood flow.

Author	Sample Size/Population	Stimulation Method	Control	Outcome	Main Results
Studies on healthy participants					
Lang et al. (2005) [21]	16 healthy right-handed male (mean age 36 ± 9.7)	Left M1, 1 mA, 10 min, anodal and cathodal	Sham stimulation	H215O positron emission tomography of regional CBF at rest and during finger movements	Anodal and cathodal tDCS induced widespread increases and decreases in rCBF in cortical and subcortical areas compared to sham; These changes persisted throughout the 50-minute period of PET scanning.
Paquette et al. (2011) [22]	9 healthy (mean age: 28 years; 3 males)	Bilateral M1 (anodal on dominant side, cathodal on non-dominant), 2 mA, 4 min	Sham stimulation	Positron emission tomography (PET)	tDCS with active movement; changes in rCBF were significantly lower on the cathodal than the anodal side compared with sham stimulation. Bilateral tDCS stimulation can induce changes in brain activation during movement and the cathode induces stronger effects.
Merzagora et al. (2010) [23]	12 healthy (mean age 29.5 ± 3.9; 6 males)	Left frontal (FP1), 1 mA, 10 min, anodal	Sham stimulation	Functional near-infrared spectroscopy (fNIRS)	Anodal tDCS significantly increased oxyhemoglobin (HbO2) concentration compared to sham stimulation; Changes lasted longer with increased stimulation time.
Giovanella et al. (2018) [22,24]	20 healthy (11 males)	Left frontal lobe (AF7) 1 mA, 10 min, anodal or cathodal	Sham stimulation	Functional diffuse correlation spectroscopy (fDCS) and time-resolved functional near-infrared spectroscopy (TR-fNIRS)	CBF increased in both anodal (10%) and cathodal (11%) stimulation but not the sham stimulation; Changes were observed in the ipsilateral hemisphere only; Increase in CBF was constant during the stimulation period and lasted at least 30 min after.
Takai H. et al. (2016) [25]	7 healthy (mean age: 22.8 ± 2.0)	Right M1, 1 mA, 20 min, anodal and cathodal	Sham stimulation	Diachronic intracranial hemodynamic changes using near-infrared spectroscopy	Both anodal and cathodal tDCS led to significantly lower concentrations of O2Hb in the contralateral premotor cortex, supplementary motor area, and M1, suggesting widespread changes in cerebral blood flow.
Zheng X. et al. (2011) [26]	14 healthy (mean age 25.7 ± 5.7; 9 males).	Right M1, average 1.4 mA, anodal and cathodal	No	Arterial spin labeling in the MRI scanner with an alternating off–on tDCS sampling paradigm	tDCS increases rCBF and is selectively based on the polarity of the stimulation, with a secondary impact from the stimulation strength; The correlation between change in rCBF and current strength was positive in anodal stimulation and negative in cathodal stimulation; Effects were seen in network activity directly beneath stimulating electrode and between functionally connected brain regions
Jamil A. et al. (2020) [27]	29 healthy (mean age 25.0 ± 4.4; 16 males)	Left M1; 0.5–1.0–1.5–2.0 mA, 15 min, anodal or cathodal	Sham stimulation	Resting-state arterial spin labeling (ASL-MRI) and compared changes to TMS-induced cortical excitability	Both anodal and cathodal tDCS showed intensity- and polarity-dependent changes in CBF compared to sham; The 2.0 mA intensity led to the greatest blood flow alterations compared to lower intensities of 0.5–1.5 mA; Changes lasted 60–75 min for 0.5–1.5 mA and entire 2 hr post-treatment evaluation period for 2.0 mA anodal tDCS; At all levels of intensity, anodal electrode led to higher changes in perfusion compared to cathode; Correlations between changes in CBF and motor-cortical excitability measured using TMS.
Mosayebi-Samani et al. (2021) [28]	29 healthy right-handed participants (mean age 25.0 ± 4.44; 16 males)	Left M1; 0.5–1.0–1.5–2.0 mA, 15 min, anodal or cathodal	Sham stimulation	Functional magnetic resonance imaging (MRI) via arterial spin labeling (ASL)	Anodal tDCS increased CBF under electrode at all intensities, mostly in the early stages; Cathodal tDCS decreased CBF, except for the 0.5 mA condition in both polarity conditions, especially in the late stages.
Shinde A. et al. (2021) [29]	32 healthy (mean age 34.2 ± 13.5; 15 males)	Unihemispheric (anode on C4, cathode on Fp1) and bihemispheric (anode on C4, cathode on C3) montages, 2 mA or 4 mA, 10 min	Sham stimulation	Functional magnetic resonance imaging (MRI) via arterial spin labeling (ASL)	CBF in the right hemispheric peri-rolandic area increased with dose under the anodal electrode, while showing trend to increase with dose and significant effect of montage in left hemispheric peri-rolandic area; The bihemispheric montage showed additional rCBF increases in frontomesial regions in the 4 mA condition but not in the 2 mA condition.
Sherwood et al. (2021) [30]	47 healthy (mean age 27.9 ± 4.85; 38 males)	Left prefrontal cortex, 1 or 2 mA, 30 min, three days, anodal	Sham stimulation	Pseudo-continuous arterial spin labeling (pcASL)	Anodal tDCS showed increased CBF in multiple regions, persist for up to 24 h following stimulation.
Liu et al. (2022) [31]	9 young healthy individuals (mean age 31.22; SD 4.55; 5 males)	Left M1, 0.5–1.0–1.5–2.0 mA, 30 s, anodal	No	Magnetic resonance imaging (MRI) combined with pseudo-continuous arterial spin labeling (pcASL MRI) in targeted left M1 hand area	pcASL MRI did not consistently show immediate effects of short-duration anodal tDCS on CBF.
Sacca et al. (2023) [32]	37 healthy females (mean age 27.4 ± 6.4)	Right DLPFC, 2 mA, 20 min, 3 consecutive days, anodal and cathodal	Sham stimulation	Cerebral blood flow using pulsed continuous arterial spin labeling (pCASL)	Anodal tDCS increased CBF in bilateral thalamus, insula, lateral prefrontal cortex, midcingulate cortex, occipital lobe, and cerebellum; Increased CBF in the right insula in both the cathodal and sham tDCS groups.
Stagg et al. (2013) [33]	24 healthy (mean age 26.7; 6 females)	Left DLPFC, 1 mA, 10 min, anodal or cathodal	Sham stimulation	Pseudo-continuous arterial spin labeling (pcASL)	Polarity-dependent CBF changes in regions structurally connected to left DLPFC (thalami) with anodal (increased) and cathodal (decreased) stimulation.
Muccico et al. (2022) [34]	23 healthy (mean age = 35.6 ± 15; 10 males)	Left anodal dorsolateral prefrontal cortex, 2 mA, 15 min, anodal or cathodal	No	Cerebral blood flow (CBF), venous blood oxygenation (Yv), and cerebral metabolic rate of oxygen (CMRO2)	Global CBF, from bilateral internal carotid and vertebral arteries, was significantly greater during stimulation and remained elevated post-tDCS; Increased CMRO2 levels with CBF during tDCS and remained increased post-tDCS; interconnection between the neuronal and hemodynamic properties; Venous blood oxygenation levels were similar during tDCS and post-tDCS.
Stefano et al. (2022) [35]	11 right-handed healthy (mean age 31 ± 5.6; 5 males)	Right temporo-parietal junction, 1, 2, and 3 mA, anodal and cathodal	Sham stimulation	Mean middle cerebral artery blood flow velocity (MCA-BFv) bilaterally using transcranial doppler ultrasound during and after stimulation	None of the tDCS stimulation showed significant changes in MCA-BFv in ipsilateral or the contralateral stimulation side.
Giorli et al. (2014) [32,36]	25 healthy (mean age 26.43 ± 6.63; 19 females)	Right M1, 1 mA, 15 min, anodal or cathodal	Sham stimulation	Cerebral vasomotor reserve (VMR) by transcranial color-coded sonography	tDCS induced polarity-specific alterations in VMR; Anodal tDCS decreased mean flow velocity (MFV) in MCA, whereas cathodal tDCS increased MFV; No significant changes in sham stimulation.
Razza et al. (2022) [37]	23 healthy (mean age 28.7 ± 7; 15 females)	Factorial 2 × 2 design tDCS and iTBS, 2 mA, 4 sessions (1 per week), 20 min, anodal or cathodal	Sham stimulation	Single-Photon Emission Computed Tomography (SPECT)	Polarity-dependent effect; cathodal and anodal tDCS increased and decreased DLPFC CBF, respectively.
Jeong et al. (2023) [38]	5 healthy	Bifrontal tDCS over DLPFC 1 mA, 30 min, 20 sessions	No	Single-Photon Emission Computed Tomography (SPECT)	Increased right superior frontal gyrus CBF at the follow-up.
Studies on stroke patients					
Dutta A. et al. (2015) [39]	4 chronic (> 6 months) ischemic stroke patients (aged 31 to 76; 3 males)	Over Cz, ON–OFF 30 s repeated 15 times, current density of 0.526 A/m^2^, anodal	Sham stimulation	Changes in oxyhemoglobin (HbO2) and deoxyhemoglobin (Hb) concentrations using near-infrared spectroscopy (NIRS)	Rapid alterations in regional tissue concentrations of oxygenated (HbO2) and deoxygenated (Hb) hemoglobin within the first 60 s with anodal tDCS.
Iyer et al. (2019) [40]	20 chronic stroke patients (aged 45–72 years; 13 male)	M1 (lower limb), 1 mA, 15 min, anodal	Sham stimulation	Middle cerebral artery (MCA) cerebral blood velocity (CBv), cerebrovascular resistance (CVRi), and other cerebral hemodynamic-related variables using transcranial doppler	No change was found in CBv with anodal tDCS; At baseline, responders demonstrated lower corticomotor excitability, lower CBv, and higher CVRi compared to non-responders.
Estelle Pruvost-Robieux et al. (2021) [19]	45 acute ischemic stroke patients in the MCA territory, NIHSS score between 4 and 25, and eligible for reperfusion therapies	Affected M1, 1.5 mA, 20-min epochs, delivered every hour over 6 h period, cathodal	Sham stimulation	Infarct growth (primary outcome), arterial recanalization	Cathodal tDCS is safe and feasible in acute MCA-territory stroke; No significant difference between active and sham groups. Potential benefits of C-tDCS in patients with NIHSS >10 or large vessel occlusion.
Klomjai et al. (2022) [41]	82 eligible acute stroke participants	Ipsilesional M1, 1.5 mA, 20 min for 5 consecutive days, anodal, cathodal, bihemispheric	Sham stimulation	Cerebral mean blood flow velocity (MFV) using transcranial color-coded Doppler in each MCA	None of the groups showed significant changes in the MFV in the lesioned or non-lesioned hemispheres post-intervention or at 1 month; Significantly greater clinical improvement in bihemispheric group.
Bahr-Hosseini M. et al. (2023) [20]	10 acute ischemic stroke (ineligible for reperfusion therapies) patients within 24 h from onset (7 active, 3 sham). Mean age 75 ± 10; 6 females	6 predefined montages according to the location of the large vessel occlusion, 3 + 3 dose escalation plan, cathodal	Sham stimulation	(1) Improved perfusion (reduction in hypoperfusion region volume); (2) collateral enhancement (increase in quantified relative cerebral blood volume [qrCBV]); (3) penumbral tissue salvage (tissue at risk not progressing to infarction) at 2 to 4 h (early time point) and 24 to 30 h (late time point)	Higher rates of penumbral tissue salvage and alleviation of hypoperfused ischemic regions with active stimulation compared with sham; Enhancement of rCBV and a higher rate of early recanalization post-stimulation in active stimulation; The increase in post-stimulation qrCBV showed a dose-response trend.

## Data Availability

No new data were created or analyzed in this study.
